# Who can afford to dissent at work? The mediating effect of organizational socialization on the relationship between social capital and organizational dissent

**DOI:** 10.3389/fpsyg.2024.1390527

**Published:** 2024-09-26

**Authors:** Murat Ak, Mehmet Ali Turkmenoglu, Duygu Acar, Abdullah Ramiz Hacarlioglu, Mustafa Ozbilgin

**Affiliations:** ^1^Faculty of Economics and Administrative Sciences, Department of International Trade and Business, Karamanoğlu Mehmetbey University, Karaman, Türkiye; ^2^Faculty of Economics and Administrative Sciences, Business Administration Department, Muş Alparslan University, Muş, Türkiye; ^3^Karacabey Vocational School, Management & Organization Department, Bursa Uludağ University, Bursa, Türkiye; ^4^Independent Researcher, Kahramanmaras, Türkiye; ^5^Brunel Business School, College of Business, Arts and Social Sciences, Brunel University London, London, United Kingdom

**Keywords:** social capital, organizational dissent, organizational socialization, structural equation modeling, quantitative research

## Abstract

**Introduction:**

In today’s interconnected world, fostering a culture of constructive dissent within organizations is more important than ever. Our study sheds light on how social capital—our networks and relationships—affects employees’ ability to express dissent. This study aims to empirically examine whether organizational socialization has a mediating effect on the relationship between social capital and organizational dissent.

**Methods:**

We utilized surveys to collect data from participants. Quantitative data was collected from 240 employees within the textile in Türkiye. We used structural equation modeling through SmartPLS to test four hypotheses.

**Results:**

According to the results of the SEM, social capital positively affects organizational dissent. Similarly, social capital positively affects organizational socialization. The mediation level of organizational socialization is at the level of partial mediation on the relationship between social capital and organizational dissent. Based on the results, organizational socialization positively affects organizational dissent.

**Discussion:**

We contribute to the literature by extending social capital research by illustrating that employees’ social relationships lead to organizational socialization and organizational dissent behavior at work. The results suggest that the ability of employees to show dissent behavior is conditioned by their social capital and mediated by organizational socialization. This research is particularly relevant in sectors with hierarchical structures, where encouraging voice and participation can lead to significant advancements.

## 1 Introduction

Healthy and democratic organizations are where human conditions such as opposition and dissent are normalized, and employee voice is prioritized over silence (Erbil and Ozbilgin, 2024). However, in many organizations, employee dissent is viewed as challenging management control and command (Müceldili et al., 2021; Helens-Hart et al., 2023). Although management science promotes the humanization of work and recognition of workplaces as multi-stakeholder settings where conflict and dissent are normalized, management practice often lags, engaging in dated approaches such as management control from five decades back (Ozbilgin et al., 2022). Hence, duality prevails in organizations that consider dissent legitimate or illegitimate. In such a context, exploring the conditionality of dissent is interesting: who can afford to display dissent, and under what conditions? This paper explores whether individuals with social capital endowments can afford a higher degree of dissent in organizations in a country with a weak culture and tolerance for workplace dissent and democracy.

Despite the established importance of social capital in facilitating various positive organizational outcomes, its role in the context of organizational dissent and socialization remains underexplored. While previous studies have highlighted the influence of social capital on employee behaviors, the specific mechanisms through which social capital impacts organizational dissent and the mediating role of organizational socialization have not been thoroughly investigated. This gap in the literature necessitates an empirical examination to understand how social capital and organizational socialization interact to influence dissent within organizations. Therefore, this study aims to investigate the mediating effect of organizational socialization on the relationship between social capital and organizational dissent, addressing the critical question: How does organizational socialization mediate the relationship between social capital and organizational dissent in the workplace? By addressing this question, the research contributes to the theoretical understanding of social capital’s impact on organizational dynamics and provide practical insights for enhancing democratic practices and employee voice in organizations.

We first define the concepts of social capital, organizational dissent, and organizational socialization. Then, we hypothesize the relationships between social capital, organizational dissent, and organizational socialization to show the conditionality of dissent through an empirical investigation. Findings show that individuals with social capital can afford organizational dissent and organizational socialization mediated this relationship. We add to the existing body of knowledge on social capital by demonstrating that having strong social relationships can positively affect organizational socialization and dissent behavior within the workplace. The paper has significant considerations for policy makers in industrial psychology and human resource management to consider how to normalize dissent in organizations. Recognizing the affordability of dissent through social capital suggests that human resource management interventions could seek to improve democratic workplace relations and dissent among workers by targeting workers without social capital. We contend that organizational socialization could help dissent to be normalized at work.

## 2 Literature review

### 2.1 Social capital

Social capital is a multifaceted concept that has gained wide attention over the years and found application to various disciplines such as sociology Bourdieu (1979), economy (Fukuyama, 1995), political science (e.g., Newton, 2001), psychology (e.g., Song, 2011), business administration (e.g., Lee, 2009). Although the historical roots of the concept can be found in the works of sociologists such as Durkheim, Marx, and Tocqueville, three specific theorists played a significant role in conceptualizing social capital: Bourdieu (1979, 1980, 1986), Coleman (1988, 1990, 1993) and Putnam (1993, 1995, 2000).

The complexity of social capital has led to various definitions of the concept through different points of view. For instance, in Bourdieu’s conceptualization, as capital is the force through which social differences materialize, social capital is treated as a class issue and a private good (Bourdieu, 1986). He defines social capital as “the aggregate of the actual or potential resources linked to possession of a durable network of more or less institutionalized relationships of mutual acquaintance or recognition” (Bourdieu, 1985, p. 248). Bourdieu’s definition is important as it distinguishes between two critical elements: (1) the social relationship itself that allows a variety of actors to access resources held by their associates, and (2) the amount and quality of those resources (Portes, 2009, p. 3).

Although Bourdieu can be accepted as the first theorist to conceptualize social capital, Coleman’s work achieved a more widespread acceptance, popularized the concept, and paved the way for research. Coleman has conceptualized social capital by its function and defined it as “a variety of entities with two elements in common: They all consist of some aspect of social structures, and they facilitate certain actions of actors—whether persons or corporate actors—within the structure” (Coleman, 1988, p. 98). So, in his perspective, social capital is accepted as something that facilitates the achievement of collective goals and can be used to the advantage of members of a group or a society. Furthermore, Coleman emphasizes that individuals choose to cooperate even when they must compete for their interests (Field, 2006). So, contrary to Bourdieu, Coleman emphasized cooperation rather than competition. Thus, social capital consists of relationships established between individuals to complement human capital. In Coleman’s definition of social capital, individuals direct their social actions within obligations and expectations, information channels, and norms. Therefore, three structures that embody social capital offer ways to understand and explain social actions (Rea-Holloway, 2008, p. 15). Putnam (1993, p. 35) defines social capital as the “features of social organization such as networks, norms, and social trust that facilitate coordination and cooperation for mutual benefit.” Similarly, Leana and Van Buren (1999, p. 538) define the concept as “a resource reflecting the character of social relations within the organization which is realized through members’ levels of collective goal orientation and shared trust.” In this way, social capital creates value by facilitating successful collective action.

Over the years, social capital has been identified as a multi-dimensional concept (Hidalgo et al., 2024). The most common dimensions are grounded in the work of Nahapiet and Ghoshal (1998), who offer structural, cognitive, and relational dimensions. The structural dimension refers to the tangible and external observed social constructions (e.g., social ties and networks) used to obtain information, social support, and suggestions from others. The cognitive dimension refers to the intangible aspects that are related to resources providing shared values, attitudes, and beliefs. It represents the expressions, interpretations, and meaning systems between the parties and states the bonding force that holds the community together (Nahapiet and Ghoshal, 1998; Fischer et al., 2004). Lastly, the relational component is about the nature and quality of relationships (e.g., trustworthiness, social networking). The sources of relational social capital are embedded in relationships, such as trust among members and the reliability of individual actors (Sherif and Sherif, 2006).

Another common form of social capital is the “bonding, bridging, linking ties,” based on the proximity of linkages between different actors (Harpham et al., 2002; Kreuter and Lezin, 2002; Szreter and Woolcock, 2004; Woolcock and Narayan, 2006; Ferlander, 2007). While bonding ties refer to the ties between individuals within a homogeneous group, bridging ties refer to the network connections amongst people of heterogeneous groups. Lastly, linking ties are between individuals and groups with people or organizations in positions of authority and influence. It is also possible to see other theoretical approaches that are less common, such as positive/negative (Graeff and Svendsen, 2013), instrumental/principled (Heffron, 2001; Van Deth, 2003), horizontal/vertical (Colletta and Cullen, 2002), formal/informal (Dhesi, 2000; Ferlander, 2007; Pichler and Wallace, 2007).

Over the years, the concept of social capital has received attention in organization research as it offers a valuable contribution to managerial activities (Adler and Kwon, 2002; Spence et al., 2003). The key features of social capital in the organizational setting are identified as trust (i.e., the expectation for honest and reliable behavior; Fukuyama, 1997), norms (i.e., mutual exchange of support and benefits; Harpham et al., 2002), and network interactions (i.e., creating and expanding interpersonal relationships; Gibson et al., 2014) that developed among organizational members and across organizations. In these terms, theorists distinguish between internal and external social capital (Adler and Kwon, 2002). While external social capital refers to the networks beyond the boundaries of the organization, internal social capital is based on norms of trust and cooperation between an organization’s members, which can contribute to cohesiveness, foster collective actions, and facilitate the creation of a favorable work environment (Welch and Jackson, 2007). Internal social capital also enables managing relationships strategically and allows employees to collaborate effectively in pursuing organizational goals (Acquaah, 2011; Dato-on et al., 2018). Moreover, strong internal ties facilitate the exchange of information and knowledge with members who they trust and enable different groups to interact and develop sensitivity to each other’s problems (Kim and Cannella, 2008). Thus, social capital is vital for communication, cooperation, employee commitment, strong relationships, involvement, and mutual knowledge sharing (Andrews, 2010; Nahapiet and Ghoshal, 1998; Putnam, 2000).

### 2.2 Organizational dissent

Dissent as a vital process of organizational communication has been closely linked with the conceptualization of employee voice, which Hirschman (1970) defined as “a response to dissatisfaction in organizations” (Kassing, 1997, p. 321). It is argued that dissent is a voice where employees express their divergence from organizational or managerial concerns (Hegstrom, 1995; Kassing, 1997, 2002). Kassing (1997, 1998) as the pioneer of organizational dissent research defines the concept as “expressing disagreement or contradictory opinions about organizational practices, policies, and operations” (Kassing, 1998, p. 183). Another common conceptualization belongs to Garner (2009, 2013, 2016), who defines organizational dissent as “an interactive process that occurs as a result of one or more subordinates expressing disagreement with policies, practices, or imperatives” (Garner, 2013, p. 376).

Organizational dissent can take many forms, ranging from constructive criticism to rebellion against the functioning or established norms of the organization (Roberts, 2024). Regardless of its form, it is a natural and inevitable aspect of organizational life, which can be a mechanism for organizational learning and growth, as dissenting viewpoints can challenge the status quo (Müceldili et al., 2021). Positive outcomes of dissent include such as increased employee performance (Ng and Feldman, 2012), better problem-solving (Schulz-Hardt et al., 2006), and better decision-making (Banks, 2008; Morrison and Milliken, 2000), innovation (Pyrozhenko, 2016).

The research on organizational dissent mainly concentrates on a few key points, such as the audience to whom the dissent is directed (Kassing, 1997, 2011), the factors that influence dissent expression (Kassing, 2011), and the process of dissent (Kassing, 1997). For instance, Kassing (1997, p. 322) proposed a model for explaining the process of organizational dissent, which consists of four steps: (1) triggering effects, (2) dissent strategy selection influences, (3) dissent strategy selection, and lastly (4) expressing dissent. According to this model, triggering effects lead employees to share contradictory opinions. Typical triggering events may include dissatisfaction, poor decision-making, organizational misconduct, unethical decisions and activities, unfair performance evaluation processes, work practices and processes inefficiency, unclear or conflicting roles and responsibilities, and organizational changes (Kassing and Armstrong, 2002).

After the triggering events, employees select strategies for expressing dissent based on individual, relational, and organizational influences (Kassing, 1997). Individual influences are about employees’ values and behaviors, which are determinative in their dissent strategies and refer to “predispositions and expectations people import from outside their respective organizations, as well as how they behave within the organization” (Kassing, 1997, p. 324). Relational influences include “the types and quality of relationships people maintain within the organization,” and organizational influences reflect “how people relate to and perceive organizations” (Kassing, 1997, p. 324). These three influences also affect the strategy selection of employees and work as predictors of the communication way in sharing dissent messages (Goodboy, 2011). Relational and organizational influences provide clues about how dissent will be perceived within the organization (i.e., as constructive feedback or as adversarial by the organization), which is decisive in the employees’ choice of strategy.

The final step of the model is expressing dissent. Early research on organizational dissent focused on expressions to external audiences like industry governing bodies and the media (e.g., Graham, 1986; Near and Jensen, 1983; Stewart, 1980; Westin, 1986). However, Kassing’s (1997, 1998, 2000, 2011) work identifies three potential audiences to express dissent: supervisors, coworkers, and friends or family members outside the organization. Dissent expressed to supervisors is called “articulated” or “upward” dissent. This type of dissent refers to expressing opposing views to the supervisors and managers who may have power within the organization. As a form of dissent that targets the managerial level in the organization, it occurs when employees feel that they will be perceived as constructive (Kassing, 1997). Dissent shared with coworkers is called “latent” or “lateral” dissent. This type of dissent mainly occurs in the case of that employees feel the lack of channels and environment for sharing their opposing ideas in the organization (Kassing, 2000). Kassing (1998) argues that when employees see the risk of being perceived as adversarial within the organization, they tend to share conflicting and problematic issues with less powerful members (i.e., co-workers) rather than supervisors. Lastly, dissent expressed to others outside the organization is called “displaced dissent.” In this type of dissent, employees share problematic and negative organizational issues with people in close relationships, such as family members and intimate friends. According to Kassing (1997), this is an alternative way to decrease the risk of adversarial and retaliation, but this situation points out the employees’ psychological exit.

### 2.3 Organizational socialization

Organizational socialization is accepted as the process by which individuals move “from being organizational outsiders to being insiders” (Bauer et al., 2007, p. 707). The early discussions of organizational socialization appear in Schein et al. (1965) and Schein (1968)‘s work that has influenced much of the research to date. He defines socialization as “the process by which a new member learns the value system, the norms, and the required behavior patterns of the society, organization, or group which she/he is entering” (Schein, 1968, p. 2). Relatedly, organizational socialization is defined as “the process by which an individual acquires the social knowledge and skills necessary to assume an organizational role (Van Maanen and Schein, 1979, p. 211).” Katz (1980, p. 88) reveals this process as the “introductory events and activities by which individuals come to know and make sense out of their newfound work experiences.” During the process, newcomers gain insight into their roles and develop knowledge, attitudes, behaviors, and ways of thinking to fit in with their assigned roles and organization. The socialization process also involves the internalization of the values, norms, and culture of an organization (Inzerilli and Rosen, 1983; Klein and Weaver, 2000; Meek, 1988).

There are several conceptual approaches to socialization. The ‘Stage’ approach is well-known for splitting the socialization process into temporal phases through various steps (Feldman, 1976). The process starts before the newcomer joins the organization, described as a pre-encounter stage, and reflects the newcomers’ initial views concerning the expectations of the organization and job (Bauer and Green, 1994; Porter et al., 1976; Taormina, 1997). The next step, the encounter stage, begins as the newcomer enters the organization and meets the real organizational setting. In this stage, the newcomer tests her/his expectations and reality through observations and experiences in the organization (Bauer and Green, 1994). The adaptation stage occurs when the newcomer no longer feels like an outsider and successfully adapts to the role articulated by the organization, fits in, and starts performing (Kramer, 2010). The ‘tactics’ approach, on the other hand, examines the organization’s efforts, which are formal, structured, and institutionalized methods to socialize newcomers (Van Maanen and Schein, 1979).

Van Maanen and Schein (1979) proposed six socialization tactics that managers can employ, which exist on a continuum with considerable range between the two poles. Collective (vs. individual) socialization refers to grouping newcomers and putting them through common experiences or dealing with each newcomer individually. Formal (vs. informal) socialization is about segregating a newcomer from existing staff members during the socialization period or not clearly distinguishing them from others. Sequential (vs. random) socialization involves a fixed sequence of identifiable steps compared to a random and continually changing sequence. Fixed (vs. variable) socialization provides a timetable for the steps involved, whereas a variable process does not have a timetable. The serial (vs. disjunctive) tactic is one where the newcomer is socialized by an experienced member of the organization who serves as a role model, compared to a process where a role model is unavailable. Finally, the investiture (vs. divestiture) tactic is about providing feedback to affirm the incoming identity and characteristics of the newcomer rather than not providing any feedback.

Another approach relies on handling socialization as a learning and sense-making process. Learning, which is described as “the heart of any organizational socialization model” by Ashforth et al. (2007, p. 16), is related to newcomers acquiring a variety of knowledge content and becoming effective members of the organization (Klein and Weaver, 2000; Klein and Heuser, 2008). Here, newcomers’ learning (socialization) content refers to acquiring a way of thinking and behaving (i.e., tasks, roles, norms). Moreover, this learning process includes generating insight into the organization’s interpersonal and group relationships and the nature as a whole (Ashforth et al., 2007;17). Yet more, various scholars emphasize the importance of the insiders’ acceptance of newcomers during the socialization process (Korte and Lin, 2013; Moreland and Levine, 2002).

The literature indicates that successful socialization results in adjustment, which involves developing sufficient knowledge and clarity about the role and organization (Bauer et al., 2007; Haueter et al., 2003). Hence, it leads to positive work-related attitudes such as higher task mastery, performance, job satisfaction, and organizational commitment with lower role ambiguity, role conflict, and intentions to quit (Saks and Ashforth, 1997; Saks et al., 2007). Moreover, successful socialization provides achieving acceptance from insiders (Moreland and Levine, 1982; Ostroff and Kozlowski, 1992).

## 3 Hypotheses development

### 3.1 Social capital, organizational socialization, and organizational dissent

Leena and Van Buren (1999, p. 538) define organizational social capital as a resource reflecting the character of social relations within the organization. When trust and unity prevail, individuals can establish networks across different departments and hierarchical levels, facilitating their integration into the organization. Strong internal ties ensure that employees are more connected to the network they belong to, thereby strengthening social capital and creating advantages for both the organization and the employees (Adler and Kwon, 2002; Leana and Van Buren, 1999; Leana and Pil, 2006).

Thus, through the impact of workplace relations on organizational interaction (Sandelands and Boudens, 2000; Sias et al., 2004), Garner (2013) conceptualizes dissent as an interpersonal phenomenon. Strong, trust-based relationships foster open communication, with dense networks providing multiple channels for employees to express honest feedback. These networks enable employees to share and discuss dissenting opinions more freely, making finding support for their concerns easier. Thus, communication with coworkers and supervisors’ active participants in the dissent process- helps to socially construct the dissenter’s dissent behavior (Garner, 2013). Social capital, therefore, becomes an expression of the organization’s ability to handle conflicts that may exist between the different groups (e.g., between the owners and the employees) within an organization (Hasle et al., 2007). In other words, organizational social capital may create a communication channel that enables frequent interaction between groups, effective information sharing (Sherif and Sherif, 2006), and contributes to solving organizational problems (Nahapiet and Ghoshal, 1998; Putnam, 2000).

Specifically, Kassing (2000) suggests that having a close and higher-quality relationship with one’s supervisor leads to increased articulated (upward) dissent and decreased latent (lateral) dissent (Kassing, 2000). Articulated (upward) dissent can be considered one of several forms of upward communication. Research indicates that an employee with a good relationship with her/his supervisors is more successful in communicating his dissenting behavior directly to his manager than others (Kassing, 1997; Kassing, 2000).

Thus, social capital, consisting of supportive relationships, creates positive employee attitudes such as increased mutual trust (e.g., Roth, 2022), strong organizational commitment (e.g., Tajpour et al., 2022; Watson and Papamarcos, 2002), sensitivity to organizational problems (e.g., Ko et al., 2018). When trust is high, employees may feel safer expressing dissent, believing that their opinions will be considered and respected rather than punished. So, we predict that social capital may encourage voice and speak up, promoting a more available setting for organizational dissent. Based on this we suggest the following hypothesis.

*H1: Social capital has a significant and positive effect on organizational dissent.*

When newcomers enter organizations, they may experience high levels of uncertainty in terms of the functioning of the organization. The ambiguity of organizational settings coupled with newcomers’ lack of information requires learning about the strategic and operational dynamics (e.g., the mission, tasks, etc.), as well as learning about the social dynamics within the organization (Bacharach and Lawler, 1980; Fang et al., 2011). Such uncertainties may be reduced by using various communication channels. Social capital aids in integrating newcomers into the organizational culture by exposing them to the values, beliefs, and behaviors that are prevalent within the organization. Through social interactions, newcomers can internalize these cultural elements and align their behavior accordingly. Notably, social interactions with insiders are assumed to contribute to the integration of newcomers into the existing network structures and effective adjustment to the new environment (Bauer and Erdogan, 1998; Graen and Uhl-Bien, 1995; Katz, 1980; Korte, 2009; Saks and Ashforth, 1997). A well-connected network may facilitate the dissemination of information and the integration of new employees into the organizational fabric. Additionally, strong, trust-based relationships may encourage open communication and support, which are essential for the smooth integration of newcomers. In this regard, Korte and Lin (2013) indicate that the transformation of newcomers from outsiders to insiders is heavily influenced by the quality of the relationships the newcomer develops. Moreover, their study revealed that the relational dimension of social capital, which is concerned with the characteristics of interpersonal relationships, such as friendship, liking, trust, and respect, is the dominant dimension in the socialization process. In addition, they suggest that learning the unwritten rules, shared language, meanings, and narratives within an organization, which also reflects the cognitive dimension of social capital, is facilitated by developing good relationships.

Such a perspective, which considers the influence of social relations, opposes the dominant perspective that accepts socialization as an individual process. The individualistic perspective generally accepts that newcomers are responsible for becoming insiders (Ashforth et al., 2007; Korte, 2009; Saks et al., 2007). However, some studies emphasize the influence of social relations and network ties in the socialization process (e.g., Cooper-Thomas and Anderson, 2006; Korte, 2009; Korte and Lin, 2013). Thus, considering the social dynamics as mutually constructed relations between newcomers and insiders in the socialization process goes beyond the typical individualist approach. In other words, socialization as a learning process about fitting into the organization, is accepted as a social rather than an individual process. Thus, the relationships with insiders influence how well newcomers integrate into the organization’s social structure and what resources they develop or acquire during socialization (Jokisaari and Nurmi, 2012). These relationships offer access to critical resources such as information, advice, etc. to the newcomers they need in the learning and adjustment process. During the socialization process, these critical resources afforded to newcomers by coworkers and managers (i.e., insiders) represent the social capital. Hence, strong relationships and networks with insiders are critical for a newcomer’s effective socialization and adjustment (e.g., Livi et al., 2020; Nasr et al., 2019) Based on this, we generated the following hypothesis.

*H2: Social capital has a significant and positive effect on socialization.*

Research on organizational dissent demonstrated that the longer employees reside in organizations (tenure), the more likely they express disagreement or contradictory opinions about organizational issues; in other words, they are more likely to show dissent behavior (Kassing, 2006, 2008). The rationale behind this connection between tenure and dissent is located on the idea that employees learn the norms about when and how to express dissent effectively and appropriately over time (Croucher et al., 2019).

A line of research relates employees’ tenure to assimilation, “*the processes by which individuals become integrated into the culture of an organization*” (Jablin, 2001, p. 755). The research show that successfully assimilated employees are more comfortable expressing their emotions in the workplace (Scott and Myers, 2005), and are less likely to leave the organization (Myers and Oetzel, 2003). Hence, previous research on dissent suggests that assimilation may influence dissent behavior (e.g., Kassing and Armstrong, 2001; Kassing and DiCioccio, 2004). For instance, Kassing et al.’s work reveal that more experienced and engaged employees tend to use articulated (upward) rather than lateral and displaced dissent (Kassing, 2000; Kassing and DiCioccio, 2004; Kassing et al., 2012). Since assimilation and socialization are interrelated concepts, it is possible to accept that socialization influences dissent behavior. For instance, Kassing (2006, 2008) emphasizes the role of socialization in understanding the norms regarding expression and frequency of dissent.

Furthermore, Garner (2013) argued that dissent is a co-constructed process whereby employees construct and revisit the meaning of dissent over time. Croucher et al. (2019) also argue that learning about the norms and rules of the organization may foster dissent expression in response to organizational issues. Effective socialization leads to a deeper understanding of organizational norms, values, and practices. This increased awareness can highlight discrepancies or areas for improvement, prompting employees to voice their concerns (Morrison, 1993). Being more integrated and aware of organizational norms and practices, well-socialized employees may feel more confident and empowered to express their disagreements constructively.

Hence, studies reveal that organizational socialization enhances new employee voice behavior (e.g., Liao et al., 2022; Wu et al., 2022). Reissner et al. (2019) suggest that when newcomers learn the organization’s history, values, and organizational politics, they become more sensitive to organizational issues. Moreover, they suggest that when they learn the organizational language, employees may speak up more in organizationally socialized ways (Reissner et al., 2019). Likewise, as socialization shapes employee perceptions and behaviors, we can expect it to influence the decision-making process and the attitudes toward organizational change, which are the key triggers of dissent expression. In this regard, we suggest the following hypothesis.

*H3: Organizational socialization has a significant and positive effect on organizational dissent.*

As an adjustment process, organizational socialization has an influence on the individual, social, and work-related dynamics within an organization (Coleman, 1988). Thus, it is used as a variable that mediates the various relationships in the organizational setting. For instance, Cepale et al. (2021) focuses on the relationship between self-efficacy beliefs and turnover intentions through organizational socialization. Islam (2010) reveal that organizational socialization mediates the relationship between the organizational context (i.e., structure and climate) and knowledge sharing. Their study suggests that having greater socialization leads to more knowledge sharing. Moreover, they emphasize that when the employees socialize successfully, they tend to share their views and discuss the organization’s problems more freely (Islam, 2010, p. 37–38).

In this study, we expect that organizational socialization has a mediating effect on the influence of social capital on organizational dissent. As discussed above, social capital may support and facilitate employees’ expression of dissent. Considering the role of strong relationships and networks with insiders in the newcomers’ successful socialization and adjustment (e.g., Livi et al., 2020; Nasr et al., 2019), we can expect employees to develop better communication skills and networks, enabling them to articulate their dissent more effectively and constructively (Garner, 2015; Ashforth et al., 2007). Also, dissent is revealed as an attempt by engaged organizational members (Kassing et al., 2012) to express voice and change (Hirschman, 1970, p. 30). Socialized employees are more likely to feel a sense of belonging and commitment to the organization, which can translate into a willingness to speak up when they perceive issues or injustices (Van Maanen and Schein, 1979). Through socialization, a member of an organization may challenge the status quo by expressing contrary opinions, perceptions, goals, or beliefs about issues (Perlow and Repenning, 2009) with the support of strong relationships and networks. Thus, we hypothesize.

*H4: Organizational socialization has a mediating effect on the influence of social capital on organizational dissent.*

## 4 Methods

**Figure 1:**
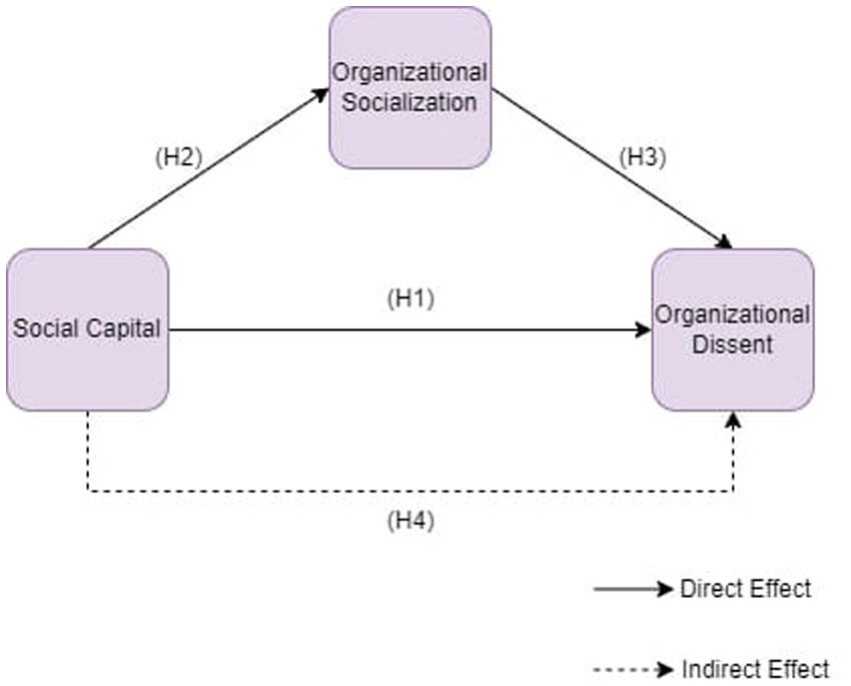
Proposed research model.

This study aims to examine the relationship between employees’ perceptions of social capital and their perceptions of organizational dissent. At the same time, whether organizational socialization mediates the relationship between employees’ perceptions of social capital and organizational dissent constitutes another objective of the study. We developed a research model (Figure 1) to examine the relationships and adopted a quantitative study to test the hypotheses. The research data have been provided by the survey obtained from a total of 240 employees from the textile sector. We chose the textile sector because long working hours and weekend shifts are frequently encountered in the textile sector. Within this intensive working context are frequent rest breaks and cafe break periods. Thus, employees are more suitable for measuring interpersonal interactions such as demonstrating dissent and socializing in these environments. In this context, it is thought that this study will be more rational to be applied in the textile sector. We adopted the maximum variation technique within the purposive sampling for this research as we assumed that we would better measure the effects of employee behaviors if we selected participants from the textile sector in which social interactions (e.g., socialization) fit our research purpose. An ethical approval received from Karamanoglu Mehmetbey University, Social and Human Sciences Scientific Research and Publication Ethics Committee to carry out this research.

### 4.1 Participants

The survey was conducted in Kahramanmaraş in Türkiye, where 49 textile factories are located. The researcher telephoned these factories to get access for their participation to the research. The researcher had some shared connections with the factory managers. Thirty factories provided permission for distributing the questionnaires. We aimed to collect at least several completed surveys from each factory to have wider perspectives on the subject. We used the drop-off pick-up method. The survey forms were delivered to managers to be distributed to participants. As a result, we received at least 8 filled questionnaires from each factory. Overall, we distributed 290 questionnaires and 259 were filled out by the participants and the response rate, which was % 89,31. However, 19 questionnaires out of 259 we received were not suitable for analysis as they had either missing responses or same patterns, hence we discarded them. Consequently, the participants of the study consisted of 240 employees. In determining the sample of the study, those working in different units and those working at the manager level based on subordinate/superior relations have been taken into consideration. The demographic characteristics of the participants are shown in Table 1.

**Table 1:** Respondents profile.

		f	%
Gender	Female	107	44.6
	Male	133	55.4
Marital status	Married	135	56.3
	Single	105	43.8
Age	18–25	19	7.9
	26–33	53	22.1
	34–41	105	43.8
	42–49	52	21.7
	50 and above	11	4.6
Educational status	High School	21	8.8
	Associate’s Degree	67	27.9
	Bachelor’s Degree	119	49.6
	Postgraduate	33	13.8
Professional seniority	1–5 Years	22	9.2
	6–10 Years	62	25.8
	11–15 Years	80	33.3
	16–20 Years	55	22.9
	21 Years and above	21	8.8
Location	Employee	165	68.8
	Manager	75	31.3

### 4.2 Data collection instrument

Survey technique has been utilized as the data collection instrument in the study. Three scales have been included in the survey form used in the research. The first of these is the “Social Capital Scale,” developed by Onyx and Bullen (2000) and translated into Turkish by Ardahan (2012). The second is the “Organizational Socialization Scale” developed by Erdoğan and Dönmez (2019). The last one is the “Organizational Dissent Scale” developed by us. The scale items used that are in the form of statements. The items coded from 1 to 5 with the lowest scale range of strongly disagree and the highest scale range of strongly agree.

### 4.3 Measuring tools

#### 4.3.1 Organizational dissent scale

For the organizational dissent scale, the scales developed by Kassing (2000) and Altınkurt and Iliman Püsküllüoğlu (2017) have been examined. With the idea that the scales in question have long statements and are not suitable for the sample content of this study, expert opinions have been consulted, and a 4-statement short-scale form has been prepared in line with the purpose of the study. For this, a scale called Content Validity Ratio / Index can be used in scale development and shortening studies based on expert opinions (Grant and Davis, 1997). Four statements with a value of more than the minimum value of 0.99 have been used in the survey form on the scale where the opinions of 5 experts have been consulted. According to Veneziano and Hooper (1997), and McKenzie et al. (1999) this value and above is sufficient for the statements in question to be included in the scale. Then, Exploratory Factor Analysis (AFA) has been applied using basic components analysis with varimax rotation to measure structure validity.

#### 4.3.2 Exploratory factor analysis

For the study, Kaiser Meyer Olkin (KMO) Sample Adequacy Criterion and Bartlett Globality Test have been used to test whether the scale developed to collect the research data is suitable for the data explanatory factor analysis and the suitability of the data. Statistical data of the KMO test and the Measure of Adequacy and the Bartlett Globality Test are shown in Table 2.

**Table 2:** KMO test statistics.

KMO sample sufficiency criterion		0.815
Bartlett sphericity test	Approximate Chi-square	335.636
	Degree of freedom	6
	*p*	0.000

When Table 2 is examined, it is observed that the KMO value of the scale has been 0.815 and the sample sufficiency for factor analysis is very good. In factor analysis, it is desirable to have a high correlation relationship between the variables and this relationship is measured by the Bartlett Globality Test. The *p* value of the Bartlett Globality Test has been found to be less than 0.05. For scale and structure validity to be acceptable, the KMO value must be greater than 0.60 and the Bartlett test must be statistically significant (Kozak, 2015, p. 150). In other words, according to the results of KMO and Bartlett tests, the sample size data set has been found to be suitable for factor analysis.

The distribution of loads for the factors of the scale is shown in Table 3.

**Table 3:** Scale loads distribution.

Factor	Eigenvalue		
	Total	Percentage of variance	Cumulative percentage
1	2.990	74.745	74.745

When Table 3 is examined, it is observed that the statement loads on the scale are collected in 1 factor, and the scale is single factor. This factor is explanatory of 74.745% of the effect to be researched.

The factor load value of the scale is shown in Table 4.

**Table 4:** Organizational dissent factor load values.

Scale items	Factor
	Organizational dissent
OO1	0.860
OO2	0.855
OO3	0.907
OO4	0.834

### 4.4 Results

#### 4.4.1 Least squares method structural equation model

PLS-SEM and CB-SEM are two different methods used in structural equation modeling. Both are used to examine relationships and structures between variables, but they have different theoretical and methodological approaches. The CB-SEM method is based on the covariance matrix between observed variables. This matrix measures the relationships between variables expected to fit a theoretically specified model. This method can sometimes have difficulty dealing with small sample sizes or complex relationships between variables (Hair et al., 2017). PLS-SEM is another structural equation modeling method widely used in social and management sciences. PLS-SEM focuses on the relationships between the components (or factors) of variables rather than the covariance between variables. This method is particularly suitable for modeling complex relationships and working with small sample sizes (Henseler et al., 2015). While CB-SEM is generally preferred for large samples or models with complex structures, PLS-SEM is more suitable for small samples or relatively less complex models (Anderson and Gerbing, 1988). While CB-SEM requires estimating the measurement and structural models separately, PLS-SEM can combine and estimate both models simultaneously. Since PLS-SEM is the most appropriate model for our research, the characteristics of our data set, and our theoretical basis made this method preferred in this study. The validity and reliability of the model were verified by checking all necessary indices in the PLS-SEM output.

#### 4.4.2 Measurement model

In evaluating the research model, the statement reliability was evaluated according to factor loads, the structure reliability was evaluated according to composite reliability, the convergence validity was evaluated according to AVE values, and the discrimination validity according to correlations were checked.

Outer Loading indicates the degree to which the statements are related to the factor. It is preferred to be above 70%, but above 40% is acceptable if an explanatory analysis is performed.

CA (Cronbach’s Alpha): This indicates the internal consistency of the measurement model. If the coefficient is above 70%, the scale’s internal consistency can be said to be at a sufficient level.

CR (Composite Reliability): It is another coefficient that indicates the reliability of the model. It is preferred to be above 70%.

AVE (Average Variance Extracted): In a structural equation model, it is a term that indicates how much of a measured construct is explained by observed variables. AVE assesses how well the variance of the variables in each structural model explains the measurement error of these variables. For the AVE value, a value of 0.50 or above is considered an adequate level of explanation (Hair et al., 2010).

When Table 5 is examined, the factor load values are between 0.711 and 0.925. In model or scale development research, it is acceptable that the factor load value is in the range of 0.50–0.60 (Hulland, 1999, p.198–199). It has been observed that the factor load value of the model is within the specified range. The Cronbach Alpha value gives internal consistency. The Cronbach Alpha value ranges from “0” to “1.” If the alpha values are less than 0.50, it is considered unreliable; if it is between 0.50–0.80, it is considered moderately reliable; and if it is more than 0.80, it is considered highly reliable (Salvucci et al., 1997, p. 115). The social capital factors of the model have a Cronbach Alpha value of 0.970; the Cronbach Alpha value of organizational dissent factors is 0.915; Cronbach’s Alpha value of organizational socialization factors is 0.951; and the model has internal consistency and provided statement reliability.

**Table 5:** Model Factor Analysis Results.

Factor	Statements	Factor load	Statement reliability	CA	CR	AVE
Social capital	SC01	0.723	0.523	0.970	0.972	0.614
	SC02	0.761	0.579			
	SC03	0.711	0.506			
	SC04	0.777	0.604			
	SC05	0.757	0.573			
	SC06	0.779	0.607			
	SC07	0.812	0.660			
	SC08	0.833	0.694			
	SC09	0.734	0.539			
	SC10	0.831	0.691			
	SC11	0.781	0.610			
	SC12	0.822	0.676			
	SC13	0.797	0.636			
	SC14	0.827	0.684			
	SC15	0.820	0.673			
	SC16	0.796	0.633			
	SC17	0.819	0.671			
	SC18	0.785	0.617			
	SC19	0.743	0.552			
	SC20	0.788	0.621			
	SC21	0.760	0.577			
	SC22	0.767	0.588			
Organizational dissent	OO1	0.907	0.823	0.915	0.918	0.798
	OO2	0.887	0.787			
	OO3	0.925	0.855			
	OO4	0.853	0.727			
Organizational socialization	OS1	0.840	0.706	0.951	0.951	0.804
	OS2	0.909	0.826			
	OS3	0.922	0.850			
	OS4	0.896	0.803			
	OS5	0.914	0.835			
	OS6	0.897	0.805			

Composite Reliability (CR) shows the reliability of the model. Composite Reliability (CR) values must be 0.70 or greater than this value (Doğan, 2019). The Composite Reliability (CR) value of the social capital factors of the model is 0.972; the Composite Reliability (CR) value of organizational dissent factors is 0.918, and the Composite Reliability (CR) value of organizational socialization factors is 0.951. It has been observed that the Composite Reliability (CR) values for all factors are over 0.70. Thus, the statements in the factors and the model have structure reliability.

For convergence validity, the AVE (Average Variance Extracted) values of the model have been examined. The AVE value must be greater than 0.50 (Doğan, 2019). The AVE value of the social capital factors of the model is 0.614; the AVE value of organizational dissent factors is 0.798; and the AVE value of organizational socialization factors is 0.804. It has been observed that the AVE value is above the specified value, and the model’s convergence validity is valid.

The dissociation validity values in the model show that the factor has a structure that is suitable for the factor to be the largest values within the row and column values to which it belongs (Fornell and Larcker, 1981). One way to check dissociation’s validity is by using HTMT rates. It is preferable that the ratio is less than 0.85 or 0.90. It can also be obtained by taking the square root of the AVE values. In cases where the HTMT rate is below 1 in the 90% confidence interval, it is accepted that the condition of dissociation validity is met (Henseler et al., 2015).

Table 6 shows the dissociation validity values according to Fornell-Larcker and HTMT ratio.

**Table 6:** Fornell-Larcker and dissociation validity by HTMT ratio.

	Social capital	Organizational dissent	Organizational socialization
According to the Fornell-Larcker criterion			
Social capital	**0.784**		
Organizational dissent	0.829	**0.893**	
Organizational socialization	0.851	0.841	**0.897**
According to HTMT rate			
Social capital			
Organizational dissent	0.870		
Organizational socialization	0.871	0.899	

The square roots of the AVEs are highlighted in bold.

When Table 6 is examined, it is observed that according to the Fornell-Larcker criteria, the factors do not have the largest values among the row and column values to which they belonged and the model do not comply with the dissociation validity criteria, but the HTMT ratios of the factors are below 1 and the model complies with the dissociation validity criteria.

#### 4.4.3 Evaluation of the structural model

After reaching the conclusion that the research model provided the reliability of matter and structure, convergence and dissociation validity, path analysis test has been applied to test the hypotheses. The research model consists of social capital, organizational dissent, and organizational socialization factors. The model of the study is shown in Figure 1. In the study, the structural model was evaluated after the conclusion that the measurement model provided reliability of matter and structure, convergence, and dissociation validity.

The VIF value is a coefficient that gives information about the presence of a multi-connection problem. If the VIF value is greater than 10, it means that the model has multiple connection problems. According to Hair et al. (2014) VIF values above 5 indicate a multi-connection problem. In our research, it has been concluded that the VIF values are between 2,472 and 4,974 and that there are no multiple connection problems. For the SRMR value, values below 0.08 are defined as good fit values, while an SRMR value of 0 indicates perfect fit. In our research, SRMR value has been found to be 0.075 and it has been concluded that it has a good compliance value. The hypotheses developed based on the structural model of the research have been tested.

T Value: This value indicates whether the indicators that make up each factor are statistically significant. If greater than 1.96, the indicator is significant for the factor.

VIF Value: It is a coefficient that gives information about the presence of multicollinearity problem. If the VIF value is greater than 10, there is a multicollinearity problem in the model.

The path model results show whether the established relationships are also supported by the model. When Table 7 is examined, the T values of the path coefficient values for the H1, H2, H3, and H4 hypotheses are greater than the T table value of 1.96 specified in the literature at a confidence interval of 95%. In addition, it has been observed that the *p* values of the road coefficient values for the H1, H2, H3, and H4 hypotheses are less than 0.05. It has been concluded that H1 “Employees have a significant and positive effect between their perceptions of social capital and their perceptions of organizational dissent,” H2 “Employees have a significant and positive effect between their perceptions of social capital and their perceptions of organizational socialization,” H3 “Employees have a significant and positive effect between their perceptions of organizational socialization and their perceptions of organizational dissent” are supported.

**Table 7:** Path coefficients and test results for hypotheses.

H. No	Path	Path coefficient	t	*p*	Assessment
H1	Social capital → organizational dissent	0.829	37.485	0.000	Supported
H2	Social capital → organizational socialization	0.851	48.295	0.000	Supported
H3	Organizational socialization → organizational dissent	0.489	7.040	0.000	Supported
H4	Social capital → organizational socialization → organizational dissent	0.416	6.885	0.000	Supported

### 4.5 Mediating effect

In our structural model, we have three variables: social capital, organizational dissent and organizational socialization. Based on the literature, organizational socialization was modeled as a mediator to address the research question of whether organizational socialization mediates the relationship between social capital and organizational dissent. To test the mediating effect, bootstrapping (5,000 bootstraps were used) was utilized within the scope of PLS-SEM (Hair et al., 2014). Following this path, the study first examined the significance level of the direct effect without organizational socialization, which is a mediating variable, by using the bootstrapping process in SmartPLS. Then, the mediating variable, organizational socialization, was included in the model and the significance level of the indirect effect was examined with path coefficients and relevant t values.

One of the methods used to calculate the mediation effect is the Variance Account For (VAF) value (Hair et al., 2014). The VAF value is found from the indirect effect/total effect formula, and 0.80 and above indicates that there is a full mediating effect, 0.20–0.80 indicates that there is a partial mediating effect, and a value below 0.20 indicates that it does not constitute a mediation effect (Hair et al., 2013). It has been concluded that hypothesis H4, “Organizational socialization has a mediating effect on the effect of employees’ perceptions of social capital on their perceptions of organizational dissent,” has been supported. The mediating effect of organizational socialization on the effect of social capital on organizational dissent has been found to have a VAF value of 0.501 and it has been concluded that there has been a partial mediation effect.

## 5 Discussion and conclusion

This research aimed to investigate the mediating effect of organizational socialization on the relationship between social capital and organizational dissent. As a result of the analyses on the relationship between social capital and organizational dissent, it has been concluded that social capital has a significant and positive effect on organizational dissent. This implies that when employees have supportive relationships with their supervisors, they are more likely to demonstrate their dissenting behavior. Furthermore, having H1 accepted, we contribute to the literature by concluding that quality relationships of employees provide a setting for their objections, and -to our knowledge- previous research has not examined this relationship yet. Second, we found that social capital is positively associated with organizational socialization. This result suggests that social relations and network ties are quite influential in the socialization process, supporting previous studies (Cooper-Thomas and Anderson, 2006; Korte and Lin, 2013).

Third, we examined the relationship between organizational socialization and organizational dissent. Our results suggest that organizational socialization has a significant effect on organizational dissent. Based on this result, organizational socialization enhances new employee voice behavior. In other words, when newcomers get to know the organization and its language, they tend to speak up more in the organizationally socialized ways. These results align with previous research (e.g., Reissner et al., 2019; Liao et al., 2022; Wu et al., 2022). Finally, we found a partial mediation effect of organizational socialization on the relationship between social capital and organizational dissent, and H4 has been accepted. This implies that having the role of strong relationships with insiders in the newcomers’ successful socialization mediates the relationship between social capital and organizational dissent. This finding is supported by prior research such as Livi et al. (2020); Nasr et al. (2019).

As a theoretical implication, we contribute to the literature by extending social capital research by illustrating that strong social relationships of employees can lead to organizational socialization and organizational dissent behavior at work. Although research on social capital has grown over the last decades (Lee, 2009), its consequences, such as organizational dissent and socialization, remained underexplored. Our study is an empirical response to the calls of social capital research (Adler and Kwon, 2002) to enhance this line of work, i.e., the impact of social capital on employees’ behavior.

Bourdieu (1987) argues that individuals from the margins (heterodoxy) clamor for inclusion, which is often blocked by the inside track (orthodoxy). However, for social progress to happen, legitimate outsiders (heterodox members) should be included. Therefore, organizational dissent, i.e., the inclusion of heterodox viewpoints, is important for innovation, creativity, and general progress in organizations.

The findings have significant implications for organizational policy and practice. Organizational dissent is an important marker of workplace democracy. Findings suggest that workers’ ability to show dissent is conditioned by their social capital and mediated by organizational socialization. Widening humanization and democratization of work require policymakers and human resource professionals to adopt organizational socialization interventions that create safe spaces for dissent.

However, the application of these variables in different sectors and samples may be recommended to future researchers to obtain comparable results and to improve the literature richness of the subject. The study covers only a certain segment of the employees operating in a certain sector in a province, and the survey forms filled out by a limited number of employees in a province have been considered in the study. In this context, the research reveals the ideas, perceptions and attitudes of the employees who participated in the survey in only one province. Future research may examine social capital and its effects in other sectors such technology sector employees. Future researcher may use longitudinal data to compare behaviors of employees.

## Data Availability

The raw data supporting the conclusions of this article will be made available by the authors, without undue reservation.
